# Antioxidant Activities of Polyphenols Extracted from *Perilla frutescens* Varieties

**DOI:** 10.3390/molecules14010133

**Published:** 2008-12-31

**Authors:** Linghua Meng, Yves F. Lozano, Emile M. Gaydou, Bin Li

**Affiliations:** 1 CIRAD, UMR GPEB Génie des Procédés, Eau, Bioproduits, TA 40/16, 73 avenue J.F. Breton, 34398 Montpellier cedex 5, France; 2 UMR CNRS 6263, Equipe AD2M (Phytochimie), Institut des Sciences Moléculaires de Marseille, Université Paul Cézanne, Faculté des Sciences et Techniques de Saint-Jérôme, Avenue Escadrille Normandie Niémen, Case 461, Marseille cedex 20, France; 3 South China Agricultural University Wushan, Tianhe, 510642 Guangzhou, P.R. China.

**Keywords:** *Perilla frutescens*, Polyphenolics, Anthocyanins, Flavonoids, Cinnamic acid derivatives, Antioxidant activity, DPPH.

## Abstract

Various cultivars of *Perilla frutescens* (L.) (var. *crispa* and var. *frutescens*) Britt. were harvested in China and Japan. They were easily differentiated on the basis of their foliage color, that varied from red to green. Water extracts of dried plants were investigated for their antioxidant activity (AA) and their polyphenolic compounds compared. Among them, cinnamic acid derivatives (coumaroyl tartaric acid, caffeic acid and rosmarinic acid), flavonoids (apigenin 7-*O*-caffeoylglucoside, scutellarein 7-*O*-diglucuronide, luteolin 7-*O*-diglucuronide, apigenin 7-*O*-diglucuronide, luteolin 7-*O*-glucuronide, and scutellarein 7-*O*-glucuronide) and anthocyanins (mainly *cis*-shisonin, shisonin, malonylshisonin and cyanidin 3-*O*-(*E*)-caffeoylglucoside-5-*O*-malonylglucoside) were quantified. AA assays are based on the inhibition of the free radical 2,2-diphenyl-1-picrylhydrazyl (DPPH). The DPPH radical scavenging activity was calculated as Trolox^®^ [(±)-6-hydroxy-2,5,7,8-tetramethylchromane-2-carboxylic acid] equivalent antioxidant capacity (TEAC). The mean amount of total phenolics of the water extracts (4-29 µmol/100 mL) and the TEAC value calculated (23-167 µmol TE/100 mL) confirmed the high antioxidant activity of these leaf water extracts. These results were highly correlated within some *o*-dihydroxylated polyphenolic compounds and AA.

## Introduction

**Figure 1:**
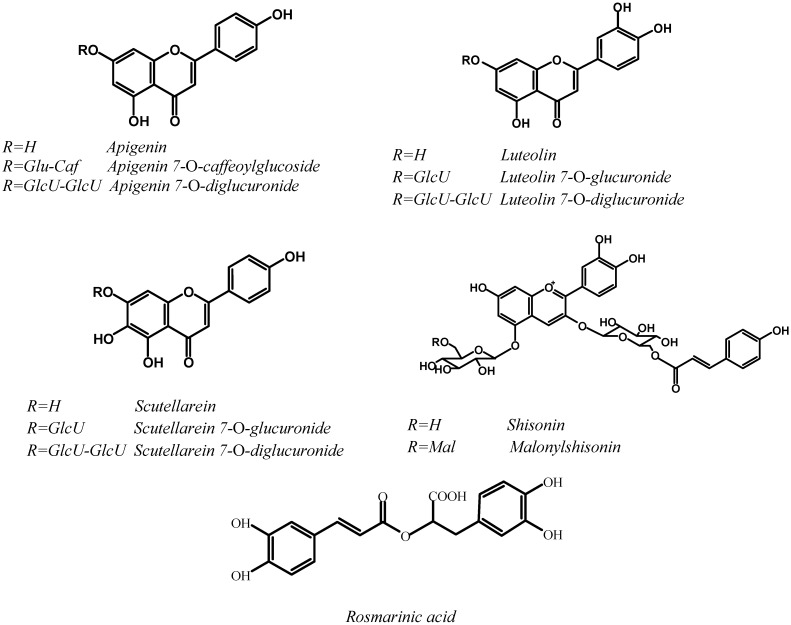
Chemical Structures of major Polyphenolic Compounds in *P. frutescens* Leaves.

*Perilla frutescens* (L.) Britt. (Lamiaceae) is an edible plant frequently used in Asian countries such as China, Korea and Japan [1]. Among the varieties, two which are traditionally used by local people are generally grown (var. *frutescens*, and var. *crispa*). Leaves of *P. frutescens* var. *frutescens* are used as a fresh vegetable and to process pickles, whereas *P. frutescens* var. *crispa* is more often used in China for its medicinal properties. Chemotypes can be found within these two *Perilla* varieties, which may be differentiated by their different leaf and stem colors, which vary from green and red and up to purple, indicating the occurrence of anthocyanins. It has been shown that the red color is mainly due to the presence of malonylshisonin (3-*O*-(6-*O*-(*E*)-*p*-coumaryl-β-D-glucopyranosyl)-5-*O*-(6-*O*-malonyl-β-D-glucopyranosyl)-cyanidin) [2, 3] (Figure 1). In the case of green-leaf chemotypes, the content of anthocyanin type compounds must be low, which in turn should affect bioactivity. *P. frutescens* are not only used as food ingredients but also for skin creams, soaps, and medicinal preparations, because of their recognized bioactivities, such as antioxidant [4, 5], anti-allergic [6, 7], anti-inflammatory [8], and anti-HIV-1 activity [9]. 

The present study aimed to evaluate the total polyphenolic contents (cinnamic, flavonic and anthocyanic derivatives), and the antioxidant capacities of eight water extract samples of *P. frutescens* varieties belonging to the var. *frutescens*, and var. *crispa*, and collected from various growing areas including China and Japan. The antioxidant activities (AA) of these two varieties were compared and correlations between AA and the various phenolic families determined. While several methods have been developed to monitor the total antioxidant capacity in biological samples [10,11,12, 17,18,19], we have used the DPPH (2, 2-dipheny-l-picrylhydrazyl) radical assay to evaluate antioxidant activity and expressed the AA in TEAC (Trolox^®^ equivalent antioxidant capacity).

## Results and Discussion

Eight leaf samples of *P. frutescens* (Table 1) were collected from various area of China (seven samples) and one sample from Japan. The leaves showed different degrees of red to green colorations, the *crispa* variety being exclusively red. Water-soluble polyphenolics were extracted from the dried leaves and analyzed by DAD-HPLC. The red type samples, **1-4**, gave similar DAD-HPLC chromatograms at 530 nm, with six peaks which have been identified as anthocyanins [3]. The total anthocyanin content varied from 2.9 to 4.0 µmol/100 mL (expressed as cyanidin equivalent). Malonylshisonin was the major anthocyanin in all *P. frutescens* red samples, followed by shisonin. A very small amount of anthocyanin compound was detected by DAD-HPLC in **5**, but not in the other green samples. 

Water extract samples quantitatively analyzed by DAD-HPLC at 325 nm, showed nine compounds, including three phenolic acids (coumaroyl tartaric acid, caffeic acid and rosmarinic acid) and six flavones (apigenin 7-*O*-caffeoylglucoside, scutellarein 7-*O*-diglucuronide, luteolin 7-*O*-diglucuronide, apigenin 7-*O*-diglucuronide, luteolin 7-*O*-glucuronide, and scutellarein 7-*O*-glucuronide). We observed that the number of phenolic compounds, other than anthocyanins, detected in both red green and red type *P. frutescens* cultivars was higher than in the green type ones (Table 1). Total cinnamic derivatives content, varied from 5 to 11 µmol/100 mL and total flavones content varied from 12.0 to 18.5 µmol/100 mL in the case of red type samples. 

The antioxidant activity (AA) was determined for each sample and compared to their polyphenolic compound contents, as shown in Table 1. AA assays employed the inhibition of free radical 2,2-diphenyl-1-picrylhydrazyl (DPPH) test/method which is one of the oldest and most frequently used method for total antioxidant potential/capacity of food extracts [10,11,12]. It is based on the ability of an antioxidant to give hydrogen radical to synthetic long-lived nitrogen radical compound DPPH. A blue-violet color changes gradually to green and yellow (absorption maximum at 405 nm), and a decrease in absorbance at 517 nm is monitored.

**Table 1:** Antioxidant Activity (µmol TE/100 mL) and Polyphenol contents (µmol/100 mL) in *Perilla frutescens* water extracts.

Sample	Fresh leaf color	AA^a^ O	Anthocyanins^ b^	Cinnamic derivatives		Flavone derivatives^ c^			Total polyphenols
				Caffeic acid	Rosmarinic acid	Luteolin derivatives	Apigenin derivatives	Scutellarein derivatives	
**1**	Red	135	3.0	3.0	7.0	4.9	4.8	5.9	28.6
**2**	Red	114	3.3	3.3	7.7	5.1	6.8	2.6	28.8
**3**	Red-green	167	2.9	2.6	7.4	6.2	3.5	2.3	24.9
**4**	Red-green	148	4.0	2.0	2.0	5.9	6.8	5.8	27.5
**5**	Green	46	0.5	n.d.	0.1	1.3	2.3	0.6	4.8
**6**	Green	103	n.d.^ d^	2.1	1.8	0.5	2.9	1.5	8.8
**7**	Green	26	n.d.	0.1	n.d.	0.6	4.2	0.6	5.5
**8**	Green	23	n.d.	0.1	n.d.	0.4	2.6	0.5	3.6

^a^ Antioxidant Activity (AA), means of triplicates; ^b^: *cis*-shisonin, shisonin, malonylshisonin and cyanidin 3-*O*-(*E*)-caffeoylglucoside-5-*O*-malonylglucoside, expressed as cyanidin equivalent; ^c^: apigenin 7-*O*-caffeoylglucoside, apigenin 7-*O*-diglucuronide, luteolin 7-*O*-diglucuronide, luteolin 7-*O*-glucuronide, scutellarein 7-*O*-glucuronide and scutellarein 7-*O*-diglucuronide, expressed as luteolin 7-*O*-glucoside; ^d^: not detected

The bleaching action is mainly attributed to the presence in the solution of antioxidant compounds like polyphenols. The amount of inactivated DPPH° is proportional to the concentration of added flavonoids [13], thus, the classical calibration procedure based on the use of Trolox^®^ as standard, can be applied for AA quantification [14,15,16].

As it can be seen in Table 1, the content of phenolic compounds and the AA are partly correlated with the foliage color of the *P. frutescens* varieties considered. Water extracts **1** to **4**, obtained from the red-green and red leaf color *P. frutescens* varieties, contain anthocyanins, cinnamic acid derivatives and flavonic compounds. The total polyphenols ranged from 22 up to 30 µmol/100 mL. The AA, expressed in TEAC, is high (114-167 µmol TE/100 mL). Determination of polyphenols in water extracts of green leaf samples **5** to **8** show a relatively low content in total polyphenols (1.6-8.7 µmol/100 mL), therefore AA are poor for extracts **5**, **7** and **8** (46, 26, and 23 µmol TE/100 mL respectively). In the case of extract **6**, the AA which is 103 µmol TE/100 mL, is explained by the high content (3.9 µmol/100 mL) in cinnamic acid derivatives (mainly caffeic and rosmarinic acids). These two acids contain in their structural formula, an *ortho*-dihydroxyphenyl moiety (one and two respectively, see Figure 1), which explains the high antioxidant power.

Correlations between each polyphenolic family and corresponding AA have been investigated (Table 2). These correlations are significant at P<0.05, but not in the case of apigenin derivatives (Figure 1). This may be explained by the absence in apigenin of an *o*-dihydroxyphenyl moiety. In the case of rosmarinic acid, the molecule of which contain two *o*-dihydroxyphenyl moieties, we observed the higher correlation (R^2^= 0.85)

**Table 2:** Correlations between Antioxidant Activity (TEAC) and the main Polyphenolic compounds identified in *Perilla frutescens* leaf water extracts.

Compound		R^2^	Regression formula ^a, b^	*P*
	Anthocyanins	0.3248	y=21.38x+62.80	0.0491
**Cinnamic derivatives**				
	Rosmarinic acid	0.8477	y=14.63x+58.58	0.00002
	Caffeic acid	0.3811	y=52.21x+46.30	0.0324
**Flavone derivatives**				
	Luteolin derivatives	0.4765	y=24.39x+34.79	0.0130
	Apigenin derivatives	0.2915	y=20.89x+26.21	0.0700
	Scutellarein derivatives	0.4326	y=26.43x+42.68	0.0201
**Total polyphenols**		0.8092	y=6.21x+8.32	0.00007

^a^: y=µmol TE/100 mL of water extract ^b^: x= mol of polyphenols/100 mL of water extract

## Experimental

### General

All reagents were of analytical grade. Cyanidin chloride and rosmarinic acid were purchased from Sigma-Aldrich, 2,2-dipheny-l-picrylhydrazyl radical (DPPH) and Trolox^®^ [(±)-6-hydroxy-2,5,7,8-tetramethylchroman- 2-carboxylic acid] were supplied by Fluka and luteolin-7-*O*-glucoside by Extrasynthese.

### Plant material and polyphenolic extraction

Two samples with red fresh leaf color of *P. frutescens* (L.) Britt. var. *crispa*, were collected during 2005 in China (Liaoning area, sample **1**) and in Japan (sample **2**). Two samples with red-green fresh leaf color of *P. frutescens* (L.) Britt. var. *frutescens* were collected in China (Guangdong area, samples **3 **and 4). Four samples with green fresh leaf color of *P. frutescens* (L.) Britt. var. *frutescens* were collected in China (Shanghai, Yunnan and Fujian area, samples **5, 6 **and **7**, and **8**, respectively). 

Every dried leaf sample (1g) was extracted by diffusion, at room temperature for 4 hr, in 100 mL deionized water (1%, w/v), and acidified with 0.01 mol∙L^-1^ H_2_SO_4_ as previously described [3]. The aqueous solution obtained was extracted three times with butanol (50 mL), using a separatory funnel. The three alcoholic phases which contain all the polyphenol compounds were combined. The butanol extract was then brought to dryness using a rotating evaporator at 35 °C. The dry matter obtained was dissolved with deionized water (50 mL) and extracted with ethyl acetate (3x30 mL). The organic layer contained the phenolic acids, while the remaining aqueous phase contained anthocyanins and flavones. The ethyl acetate phase was dried under vacuum and the residue dissolved in a minimum volume of MeOH before compound fractionation, using an open-column (16 mm x 220 mm) filled with silica gel 100-RP 18 (14 g, particle size=0.040-0.063 mm, Sigma). The mobile phase was a gradient of sequential mixtures of acetonitrile/water. Presence of polyphenolics was checked by UV-Vis spectrophometry at 530 and 325 nm. The phenolic acids content of the dried ethyl acetate phase were separated using the same open-column. Anthocyanins were identified as previously described [3].

### HPLC-DAD analysis

An Agilent series 1100 HPLC instrument (Agilent, France) equipped with a quaternary pump, a diode array detector and an autosampler was used for analyses. Samples were separated on a C_18_ column (Satisfaction column, 250×4.6 mm, 0.45 µm, Cil Cluzeau, France). The mobile phase consisted of a binary solvent system and was composed of a linear gradient of A (formic acid/water, 0.5/99.5 v/v) and B (formic acid/acetonitrile 0.5/99.5 v/v). The linear solvent started from 95% A - 5% B, up to 60% A – 40% B within 60 min, at 0.8 mL∙min^-1^. The chromatograms were recorded at 325 and 280 nm. The results were expressed as rosmarinic acid equivalent for cinnamic derivatives, luteolin-7-*O*-glucoside for flavones derivatives and cyanidin equivalent for anthocyanin derivatives.

### Antioxidant Capacity Evaluation

A stock solution of DPPH radical (0.5 mmol∙L^-1^) in methanol was prepared. The solution was diluted in methanol (60 µmol∙L^-1^, approximately) by measuring an initial absorbance of 0.62 at 517 nm (room temperature). An aliquot (100 µL) of each water extract sample was mixed with DPPH/MeOH diluted solution (3.9 mL) and the absorbance decrease as the mixture was kept at room temperature in darkness was monitored. For each sample a methanol blank was also measured. The absorbance was measured at the reaction start (time zero), and at every 5 min during the first 20 min of the kinetic and then at continuous intervals of 10 min up to constant absorbance. All experiments were done in triplicate. Trolox^®^ [(±)-6-hydroxy-2,5,7,8-tetramethylchroman-2-carboxylic acid] was used as standard antioxidant. A Trolox^®^ calibration curve was established using ten Trolox^®^ standard solutions (0.2 mmol∙L^-1^ to 2 mmol∙L^-1^ prepared from the stock solution. The radical scavenging activity of each sample was calculated by the DPPH inhibition (absorbance decrease) according to the following equation [13]:

DPPH-_scavenging activity_ (%) = 100(A_blank_ - A_sample_)/A_blank_ (with A: absorbance)

The radical scavenging activity was then expressed as µmol of Trolox^®^ equivalent per 100 mL of each sample [14,15,16].
